# Lactate Monitoring using Fluorescence with Stable Boronic Acid-Functionalized Nanoparticles from Polymerization-Induced Self-Assembly (PISA)

**DOI:** 10.1021/acs.langmuir.6c01033

**Published:** 2026-05-11

**Authors:** Morvarid H. Balouchi, Zixiao Liu, Fumi Ishizuka, Joseph C. Bear, Hachemi Kadri, Per B. Zetterlund, Fawaz Aldabbagh

**Affiliations:** a Health, Education and Society, Knowledge Exchange and Research Institute (HES KERI)and School of Life Sciences, Pharmacy and Chemistry, 4264 Kingston University, Penrhyn Road, Kingston upon Thames KT1 2EE, United Kingdom; b Cluster for Advanced Macromolecular Design (CAMD), School of Chemical Engineering, The University of New South Wales (UNSW), Sydney, New South Wales 2052, Australia

## Abstract

*L* -Lactate is elevated in various disease states, and monitoring is important to the food industry and to the performance management of high-endurance athletes. Commercial monitors are enzyme-based with low upper limits of detection (<50 mM) and are less robust than synthetic sensors using *L* -lactate binding to boronic acid (BA). The fluorescence indicator displacement assay (FIDA) for lactate has not been investigated and is convenient since BA is known to bind to catechol dyes, including Alizarin Red S (ARS) with large increases in fluorescence intensity. Herein, reversible addition–fragmentation chain transfer (RAFT) dispersion PISA of *N*-phenylacrylamide (PhA) in water/ethanol using BA-containing polyacrylamide macroRAFT gave stable spherical core–shell nanoparticles (NPs) functionalized with BA at the surface. It is demonstrated that the degree of polymerization (*DP*) of the BA-containing block of NPs can be acquired from the fluorescence-derived binding constant (*K*_ARS_) using the Benesi–Hildebrand approach. FIDA involved the displacement of ARS by the *L* -lactate quencher from the fluorescent NP complex and allows the detection of higher *L* -lactate concentrations (4.0–625 mM) than current commercial monitors. The derived Stern–Volmer constant (*K*_sv_) is independent of *DP* of the BA-containing block and increased by ∼2.5 times by addition of ethanol to the pH 7.4 aqueous dispersion. When using *D* -glucose and *D* -fructose as quenchers, *K*_sv_ is about 4-fold smaller and 13-fold greater, respectively, than *L* -lactate.

## Graphical abstract

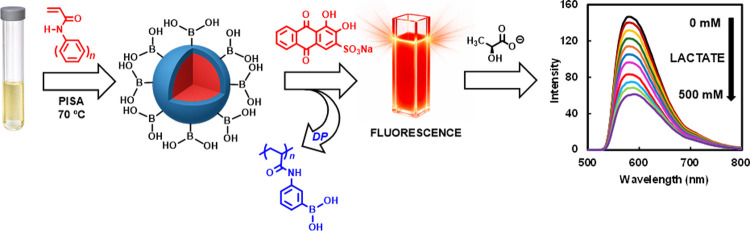

## Introduction

*L-*Lactate (LA) is a key metabolite, signaling molecule, and product of anaerobic glycolysis.^1–3^ Once associated with fatigue, as a gluconeogenic precursor, LA is now viewed by athletes as an energy source, which is required to improve performance.^3,4^ In healthcare, abnormal LA levels are an indicator of various disease states,^2,5–7^ and is important to the food industry as an additive or indicator of spoiling.^6^ Commercial LA monitors are enzyme-based, using *lactate dehydrogenase* in a spectrophotometric assay and *lactate oxidase* in an electrochemical system.^6–8^ Enzymes are however susceptible to denaturing with limited shelf-lives and have an upper limit of detection of 25–50 mM. These biosensors can accurately measure blood LA but are less reliable at high concentrations, such as in noninvasive measurements of LA in sweat that can reach >100 mM during strenuous exercise,^10,11^ hence the requirement for robust nonenzyme synthetic monitors for accurate LA detection without sample dilution.^7–9^

Boronic acid (BA)-functionalized organic and supramolecular compounds and polymers have been used in detection of *cis*-diols,^12^ including catechol dyes,^13–19^ saccharides,^14–20^ and bidentate ligands of α-hydroxycarboxylic acids, e.g. LA.^21–23^ Anslyn and co-workers developed colorimetric and fluorescence indicator-displacement assay (FIDA) for determining enantiomeric excess (*ee*) using chiral boronate ester complexes.^21^ FIDA protocols have however not been used in detecting and measuring lactate. Lowe and co-workers detected LA using holographic sensors prepared from hydrogel films derived from UV-initiated polymerization of BA-substituted-*N*-arylacrylamides.^22^ There are many reports of electrochemical sensors using BA-functionalized materials for monitoring LA, particularly in sweat, where specificity is achieved through an insignificant interfering saccharide concentration.^11,23^

BA-containing amphiphilic block copolymer nanoparticles (NPs) are commonly prepared at low concentrations by a two-step process of homogeneous reversible addition–fragmentation chain-transfer (RAFT), or other reversible-deactivation radical polymerization (RDRP), followed by dialysis with water.^24–27^ The resultant core–shell NPs have the BA-moieties at the core. NP ionization through binding to LA,^26,28^ hydroxide,^24–29^ and glucose,^24–29^ converts a portion of the hydrophobic core to hydrophilic, so changing the morphology,^26,29^ or leading to smaller, more water-soluble spherical particles.^24–28^ Aqueous polymerization-induced self-assembly (PISA) however, can directly give high concentrations of NPs without the need for lengthy dialysis.^30^ We have carried out dispersion homopolymerization of 3-(acrylamidophenyl)­boronic acid (3-BAPhA)^31^ and copolymerization with *N*-phenylacrylamide (PhA)^29^ using poly­(*N*,*N*-dimethylacrylamide, DMA) as the hydrophilic macroRAFT and steric stabilizer. During PISA, the BA-moieties form the anhydride boroxine at the water-free hydrophobic core, and postpolymerization spherical NPs transition to complex nonspherical BA-aggregates upon dilution (boroxine hydrolysis) with the aqueous dispersion solvent.^29^ To develop a reliable monitoring system for quantification of analytes, the core–shell NPs need to be stable and morphology changes must not occur. We are aware of one report of NPs from PISA with the BA moieties at the shell - Li et al. prepared polystyrene NPs for use in fructose sensing using the polymerization of styrene in methanol/water with poly­(4-vinylphenylboronic acid) macroRAFT.^19^ After polymerization a FIDA was developed by binding of NPs to catechol dyes, where fructose concentrations were determined from the depreciation in fluorescence intensity. However, this approach did not measure the binding of the dye to the NPs and the contribution of the free catechol fluorophore to fluorescence is not considered.

Herein, boroxine formation and morphology changes at the core are minimized by using BA-containing polyacrylamide as the solubilized stabilizer (poly­(3-BAPhA-*b*-DMA)) and macroRAFT, which is extended with PhA in dispersion polymerizations in 2:3 water/ethanol. Benesi–Hildebrand (B–H) is an established method for determining the binding constant (*K*_ARS_) using spectroscopic measurements such as absorbance or fluorescence techniques.^13,18,32,33^ Although reported for small molecule BA-containing molecules binding to Alizarin Red S (ARS),^13,18^ the method has not been applied to polymers. It is now demonstrated that the B–H method can be used to derive the degree of polymerization (*DP*) of the BA-containing block of amphiphilic NPs. Upon formation of the nanoparticle fluorescent complex (NP•ARS), LA is used as a static quencher for competitive displacement of ARS. This FIDA allows a wide range of LA concentrations to be detected and is compared with *D*-glucose and *D*-fructose quencher monitoring.

## Experimental Section

### Materials

The synthesis of 3-(acrylamidophenyl)­boronic acid (3-BAPhA) follows literature procedures.^31^ 2-((Dodecylthiocarbonothioyl)­thio)-2-methylpropionic acid (DDMAT; TCI, > 98%) and 2,2′-azobis­[2-(2-imidazolin-2-yl)­propane]­dihydrochloride (VA-044, Wako, 97%) were used as received. *N*,*N*-Dimethylacrylamide (DMA; TCI, 98%) was used after removal of inhibitor by passing through a column of basic alumina (Acros Organics 40–300 μm, 60 Å) and *N*-phenylacrylamide (PhA, Fluorochem, 95%) was used as received. *N,N-*Dimethylformamide (DMF; VWF, HPLC-grade ≥ 99.9%), Milli-Q water (deionized), ethanol (EtOH; VWR, ≥ 99%), and diethyl ether (Et_2_O; Fisher, > 99.5%) were used directly as solvents. Molecular sieves (MS, Alfa Aesar, 3 Å) were activated before use, by placing them in a vacuum oven (Gallebkamp 1000W, 24 V) for 2 h at 200 °C. Alizarin Red S (ARS, Sigma-Aldrich, 97%), NaOH (Alfa Aesar, 98%), sodium *L*-lactate (Alfa Aesar, > 98%), *D*-(+)-glucose (Alfa Aesar, 99%), and *D*-fructose (Alfa Aesar, 99%) were used as received. Phosphate buffered saline (PBS, pH = 7.2–7.6) was prepared according to manufacturer’s (Sigma-Aldrich) instructions. One tablet of PBS is dissolved in deionized water (200 mL) to yield 0.01 M phosphate buffer, 0.0027 M potassium chloride, and 0.137 M sodium chloride at 25 °C. The PBS is at pH 7.4, which was confirmed using a calibrated pH meter.

### Nuclear Magnetic Resonance (NMR) Spectroscopy

^1^H NMR spectra were obtained on a Bruker Avance NEO 400 MHz spectrometer using *D*_6_-dimethyl sulfoxide (DMSO-*D*_6_, Goss Scientific, 99.9%), and conversion was measured using our described method.^28,29^

### Gel Permeation Chromatography (GPC)

GPC was carried on a liquid chromatography system (Agilent Technologies 1260 Infinity model) using Agilent GPC/SEC software for Windows (version 1.2; Build 3182.29519). Molecular weight distributions (MWDs) with number-average molecular weight (*M*_n_) and dispersity (*Đ*) were measured using a Polar Gel-M guard column (50 × 7.5 mm) and two Polar Gel-M columns (300 × 7.5 mm). The eluent was DMF containing LiBr (0.01 M), which was pumped through the columns at a flow rate of 1.0 mL/min at 60 °C. The instrument was calibrated using 12 low dispersity poly­(styrene) standards (MW range from 580 to 301,600 g mol^–1^). All polymers contained BA, which prevented direct analysis by GPC.^24^ As described in our previous publications,^26,28,29,31^ polymers were converted to pinacol esters to improve solubility and to reduce affinity for the GPC stationary phase. Samples were dissolved in the eluent and filtered through a PTFE membrane with 0.22 μm pore size before injection (100 μL).

### Transmission Electron Microscopy (TEM)

TEM images were obtained using a JEOL F200 TEM, apart from Run 2 and 3, which were obtained on a JEOL 1400 microscope. Both instruments operate at an accelerating voltage of 200 kV and images were recorded via Gatan CCD imaging software. All TEM samples were prepared by dropping 10 μL of diluted sample on a glow-discharged carbon-coated copper grid (Ted Pella, Redding CA). The samples were subsequently stained with uranyl acetate for 2 min and dried at room temperature.

### Dynamic Light Scattering (DLS)

DLS measurements were conducted using a Malvern Zetasizer Nanoseries (NanoZS) instrument, which measures intensity mean average hydrodynamic diameter (*D*_h_), polydispersity index (PDI), and average zeta potential (ZP). Measurements were carried out at 25 °C using a 4 mW He–Ne laser with wavelength 633 nm, and a backscattering angle of 173°. Measurements were performed in triplicate with automatic attenuation selection and measurement position. The results were analyzed using Malvern DTS 6.20 software. Malvern Panalytical Folded Capillary Zeta cell disposable cuvettes were used for ZP, and transparent Sarstedt polystyrene (10 × 10 × 45 mm, optical path length = 1 mm) cuvettes were used for *D*_h_ and PDI.

### General Polymerization Procedure

Gilson pipettes and volumetric flasks were used to make standardized solutions. All polymerization solutions were added to borosilicate glass tubes sealed with septa and stirred at 1000 rpm with a triangular flea on a purpose-built aluminum-heating block combined with a magnetic stirrer. Nitrogen from a balloon was bubbled for 15 min through the polymerization solutions while heating at 70 °C and the solution was heated for a further 1 h and 45 min at 70 °C. The solutions were sampled (20 μL) for conversion measurements before polymerization and sampled (40 μL) at the end of the polymerization for GPC and NMR (conv.) measurements.

### MacroRAFT Stabilizer Synthesis

Poly­(3-BAPhA_28_)-TTC (used in PISA Runs 1–5; Table 1) was prepared using 50/1/0.02 of [3-BAPhA]_0_/[DDMAT]_0_/[VA-044]_0_. A standardized initiator solution was prepared by serial dilution (1.18 mM) by dissolving VA-044 (95.4 mg, 0.30 mmol) in 20% aq. DMF (250 mL). DDMAT (21.4 mg, 0.06 mmol) and 3-BAPhA (0.562 g, 2.94 mmol) were dissolved in the standardized VA-044 (1.18 mM, 1.0 mL) solution and heated at 70 °C for 2 h. The polymerization (Conv. = 90%) was stopped by placing the reaction in an ice bath. The second block was added by one-pot iterative polymerization with DMA (1.8 M) using 50/1/0.05 of [DMA]_0_/[poly­(3-BAPhA_28_)-TTC]_0_/[VA-044]_0_. The diblock copolymer was precipitated in cold Et_2_O, filtered, and dried at room temperature under vacuum for 24 h to give poly­(3-BAPhA_28_-*b*-DMA_41_)-TTC (Conv. = 87%, isolated = 0.610 g). Poly­(3-BAPhA_10_-*b*-DMA_132_)-TTC (used in PISA Run 6) was prepared in an analogous manner using 12.5/1/0.04 of [3-BAPhA]_0_/[DDMAT]_0_/[VA-044]_0_ (Conv. = 100%) followed by one-pot iterative polymerization of DMA (1.8 M) using 100/1/0.06 of [DMA]_0_/[poly­(3-BAPhA_10_)-TTC]_0_/[VA-044]_0_ (Conv. = 94%, isolated = 0.841 g). Poly­(3-BAPhA_53_-*b*-DMA_54_)-TTC (used in PISA Run 7) was prepared from poly­(3-BAPhA_53_)-TTC, which was acquired using two sequential one-pot 3-BAPhA polymerizations.^28^ The second block was added using 50/1/0.05 of [DMA = 1.8 M]_0_/[Poly­(3-BAPhA_53_)-TTC]_0_/[VA-044]_0_ to give poly­(3-BAPhA_53_-*b*-DMA_54_)-TTC (Conv. = 87%, isolated = 0.453 g).

**Table 1:** Characterization of MacroRAFTs and PISA-derived NPs (Figures 1, S1, and S2)

Run	Polymer^a^	*M*_n,th_ (kg mol^–1^)^b^	*M*_n_ (kg mol^–1^)^c^	*Đ* ^c^	*D*_h_ (nm)^d^	PDI^d^	Zeta Potential (mV)^d^
–	Poly(3-BAPhA_28_-*b*-DMA_41_)-TTC	–	12.1	1.16	–	–	–
1	Poly(3-BAPhA_28_-*b*-DMA_41_-*b*-PhA_62.5_)-TTC	21.3	26.4	1.31	–	–	–
2	Poly(3-BAPhA_28_-*b*-DMA_41_-*b*-PhA_250_)-TTC	47.5	46.2	1.63	337	0.15	–7.2 ± 0.5
3	Poly(3-BAPhA_28_-*b*-DMA_41_-*b*-PhA_500_)-TTC	85.7	86.2	1.70	491	0.08	–5.1 ± 0.2
4	Poly(3-BAPhA_28_-*b*-DMA_41_-*b*-PhA_750_)-TTC	121.1	114.8	1.79	581	0.04	–4.8 ± 0.1
5	Poly(3-BAPhA_28_-*b*-DMA_41_-*b*-PhA_1500_)-TTC	232.9	140.5	2.10	1242	0.09	–5.2 ± 0.8
–	Poly(3-BAPhA_10_-*b*-DMA_132_)-TTC	–	16.1	1.25	–	–	–
6	Poly(3-BAPhA_10_-*b*-DMA_132_-*b*-PhA_500_)-TTC	89.7	104.2	1.33	209	0.17	–4.0 ± 0.7
–	Poly(3-BAPhA_53_-*b*-DMA_54_)-TTC	–	20.2	1.28	–	–	–
7	Poly(3-BAPhA_53_-*b*-DMA_54_-*b*-PhA_500_)-TTC	93.7	88.9	1.80	325	0.07	–5.1 ± 0.2

a *DP* for PISA Runs 1–7 is calculated from *M*_n_(GPC) of the macroRAFT (poly­(3-BAPhA_*x*_-*b*-DMA_*y*_)-TTC) and conversion by ^1^H NMR (100%) for the poly­(PhA) block.

b Using *M*_n,th_ = {([PhA]_0_/[MacroRAFT]_0_) x MW­(PhA)} + MW­(MacroRAFT).

c Determined by GPC in DMF (0.01 M LiBr) calibrated against linear polystyrene standards after pinacol protection of the BA moieties.

d DLS obtained upon ∼10-fold dilution of the PISA dispersion, apart from Run 1, where the gel was diluted ∼100 fold with 2:3 water/EtOH.

### Polymerization Induced Self-Assembly (PISA)

Dispersion polymerization conditions are outlined in Figure 1 for Runs 1–5, Figure S1 for Run 6, and Figure S2 for Run 7. Run 3 is provided here as a representative procedure. The standardized initiator solution was made by dissolving VA-044 (44 mg, 0.14 mmol) in 2:3 water/EtOH (250 mL) using serial dilution. The polymerization solution was prepared by dissolving PhA (0.258 g, 1.75 mmol) and poly­(3-BAPhA_28_-*b*-DMA_41_)-TTC (38 mg, 0.004 mmol) in the standardized VA-044 solution (1.29 mL, 0.54 mM). The polymerization solutions were flushed with N_2_ for 30 min at room temperature and heated at 70 °C in an aluminum-heating block for 2 h. The dispersion was cooled to room temperature and sampled for NMR, GPC, DLS, and TEM analysis. After 30 min of standing at room temperature, the remainder of the dispersion underwent serial dilution for stability and binding studies.

**Figure 1:**
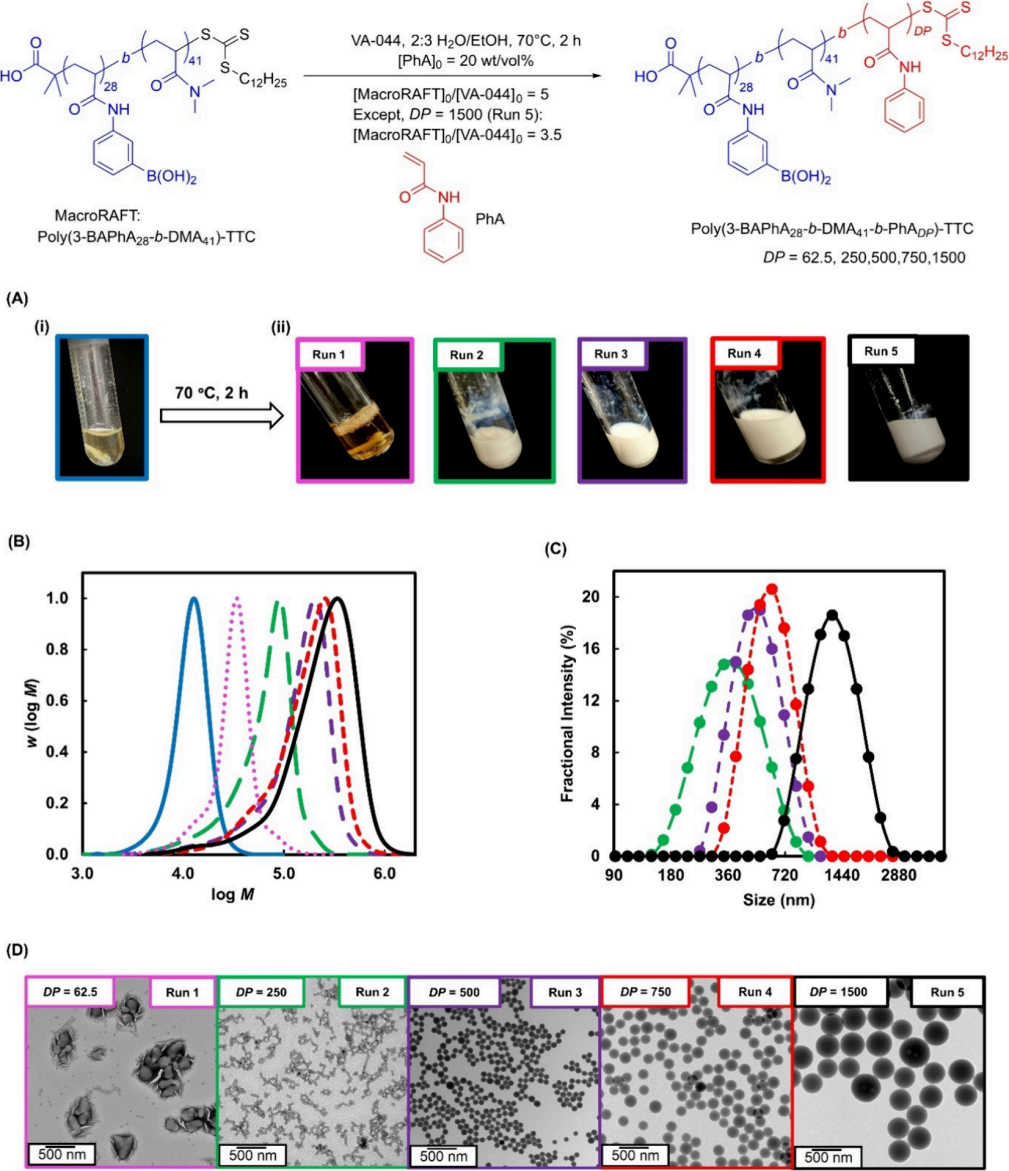
RAFT dispersion polymerizations using [PhA]_0_/[MacroRAFT]_0_ = 62.5 (pink, dotted, Run 1), 250 (green, long dash, Run 2), 500 (purple, medium dash, Run 3), 750 (red, short dash, Run 4), and 1500 (black, continuous, Run 5): (A) digital images (including stirrer bar within) (i) before and (ii) after polymerization; (B) GPC after pinacol protection of BA moieties, where the continuous blue trace is MacroRAFT (Poly­(3-BAPhA_28_-*b*-DMA_41_)-TTC); (C) DLS; (D) TEM images after 10-fold dilution, apart from Run 1 which is ∼100-fold dilution with 2:3 water/EtOH.

### Formation of Fluorescence Complex (NP•ARS)

The neat PISA dispersion with continual stirring at 1000 rpm underwent serial dilution with PBS (apart for Run 7, where 1:1 PBS:EtOH was used). ARS (17.11 g, 50 mmol) was dissolved in PBS (250 mL) by stirring for 18 h and this stock solution (0.2 mM) was further diluted for binding studies. All serial dilutions were carried out at room temperature using Gilson pipettes and volumetric flasks; however, it was difficult sampling consistent amounts of the PISA (NP) dispersions and only dilutions of ARS are deemed accurate. For example, in the analysis cell (UV or fluorescence), ARS (1.5 mL, 100 μM) was added to the NP dispersion (1.5 mL, ∼90 μM) to give the NP•ARS complex with [ARS]_0_ = 50 μM.

### UV–Vis Spectroscopy

Absorption spectra were obtained on an Agilent Cary 3500 UV–vis Compact using Fisherbrand disposable polystyrene cuvettes (4.5 mL, four-clear sided, wavelength range 340 to 750 nm) after 1 h incubation of samples at room temperature (25 °C). Analysis (Figure S4) was carried out in triplicate.

### Fluorescence Spectroscopy

Emission spectra were obtained on a Varian Cary Eclipse Fluorescence Spectrometer (wavelength range 200 to 900 nm with xenon lamp technology) using Hellma Suprasil quartz fluorescence precision cell cuvettes (80% transmission from 200 to 2500 nm). The instrument captures a data point every 12.5 ms and scans at 24,000 nm/min without peak shifts. Fluorescence analysis was after excitation at 470 nm with a path length of 1 cm. The slit width of excitation and emission was 10 nm. Analysis was carried out in triplicate.

### Preparation of Analyte (Quencher, Q) Solutions

A stock LA solution (2.5 M) was prepared by dissolving sodium *L*-lactate (11.206 g, 0.1 mol) in PBS (50 mL, 2.0 M sodium *L*-lactate). A stock glucose solution (2.0 M) was made by dissolving *D*-glucose (18.02 g, 0.100 mol) in PBS (50 mL). A stock fructose solution (0.4 M) was made by dissolving *D*-fructose (3.60 g, 20 mmol) in PBS (50 mL). Serial dilutions with PBS gave the required lower concentrations for monitoring studies. Run 7 analyte solutions and dilutions were carried out in the same manner but replacing PBS with 1:1 PBS:EtOH.

### Fluorescence Indicator Displacement Assay (FIDA)

NP•ARS (1.5 mL) was added to analyte quencher (Q, 1.5 mL) in a quartz cell and incubated for 1 h at room temperature (25 °C) prior to analysis using the Fluorescence Spectrometer (above).

## Results and Discussion

### Polymerization Induced Self-Assembly (PISA)

The macroRAFT and steric stabilizer for PISA, poly­(3-BAPhA_28_-*b*-DMA_41_)-TTC (TTC = trithiocarbonate) is insoluble in the reaction mixture at room temperature, however, is soluble at 70 °C. The hydrophilic DMA block is used to solubilize the hydrophobic 3-BAPhA block. Suitable NPs for the binding assays were found from the 2 h dispersion polymerizations (20 wt/vol %) of PhA at 70 °C in 2:3 water/ethanol at targeted *DP*s of 62.5 (Run 1), 250 (Run 2), 500 (Run 3), 750 (Run 4), and 1500 (Run 5) (Figure 1). All PISA runs proceeded to completion (100% PhA consumption by ^1^H NMR) to give free-flowing white colloidal dispersions, apart from at the lowest *DP*, which was a gel with the brown coloration indicative of boroxine formation. Control/livingness is demonstrated by the shift in the MWDs to higher MW and the proximity of *M*_n_ to *M*_n,th_ (Table 1). As expected, there is an increasing low MW tail leading to *M*_n_ being significantly less than *M*_n,th_ at the highest *DP* (Run 5). Except for Run 1 resulting in the gel, all dispersions gave uniform spherical NPs that increased in size with *DP* (hydrodynamic diameter (*D*_h_) = 337–1242 nm). A larger than expected increase in particle size relative to *M*_n_(GPC) for Run 5 is noteworthy, which may indicate a complex internal structure,^29^ however this appears not to significantly affect colloidal stability during dilution and the subsequent binding studies (below). The average zeta potential (ZP) was ∼−5 mV for Runs 3–5 indicating similar hydroxyboronate formation with the lower *DP* Run 2, having a higher absolute value of −7.2 mV. Unlike previous PISA using PhA monomer that gave higher order morphologies,^29^ only spherical NPs were now obtained, presumably due to the use of a much larger BA-containing macroRAFT contributing a high hydrophilic volume fraction to the packing parameter *P*.^34^

Dispersion polymerizations of PhA using poly­(3-BAPhA_10_-*b*-DMA_132_)-TTC (Run 6) and poly­(3-BAPhA_53_-*b*-DMA_54_)-TTC (Run 7) macroRAFT were carried out under similar conditions (as above) allowing access to NPs with less and greater, respectively, LA-binding capacity. At [PhA]_0_/[macroRAFT]_0_ = 500, both PISA Runs proceeded to 100% conversion by NMR with reasonable controlled/living character, although the white dispersion of Run 6 had partial gel-like character and Run 7 was a white free-flowing dispersion (Figures S1 and S2 and Table 1). The gelation is attributed to entanglement of the long poly­(DMA) stabilizer block (*DP* = 132) used in the macroRAFT of Run 6.^29^ TEM images showed uniform spherical NPs with DLS measured *D*_h_ = 209 and 325 nm, and average ZP of −4.0 and −5.1 mV, respectively.

In our previous work, evidence of boroxine within NPs was obtained by aqueous dilution, which caused spherical NPs to transform into higher order morphologies, including lamellae, worms, and vesicles.^29^ Conversely, with the BA-moiety at the surface, NPs from Runs 2–7 remain stable, as confirmed by DLS (*D*_h_ and ZP, Table S1) measurements and TEM images (Figures S1–S3) remaining relatively similar after dilution with different amounts of the aqueous dispersion solvent.

### Formation of the Fluorescence Complex (NP•ARS)

NP binding to ARS to give the fluorescent complex NP•ARS was carried out using the PISA dispersion diluted with neutral (pH 7.4) phosphate-buffered saline (PBS) solution (Scheme 1). Under these physiological-like conditions, upon binding, the greatest changes in ARS fluorescence intensity occur,^13^ although the polymer of the largest BA-block (Run 7) required dilution with 1:1 (pH 7.4) PBS:EtOH to maintain colloidal stability. To investigate the influence of particle size, NPs from PISA Runs 3 and 5, with the same sized BA block and *DP*s = 500 and 1500 for the PhA block were selected for binding (Table 1). The assessment of shorter and longer BA-blocks was carried out using NPs with the same poly­(PhA) block of *DP* = 500, and variable poly­(3-BAPhA) blocks of *DP*s (from GPC) = 28, 10, and 53 for Runs 3, 6, and 7, respectively. For all four polymers used to form NP•ARS, the binding of ARS at the NP surface was visually apparent with the burgundy of ARS replaced by off-white or pallor coloration (Figure S4). UV–vis spectroscopy detected the formation of NP•ARS by a blue shift in absorption from λ_max_ = 520 nm of free ARS to ∼470 nm when bound to the NPs. Thus, the fluorescence emission signal for NP•ARS (below) was measured after excitation at 470 nm.

**Scheme 1:**
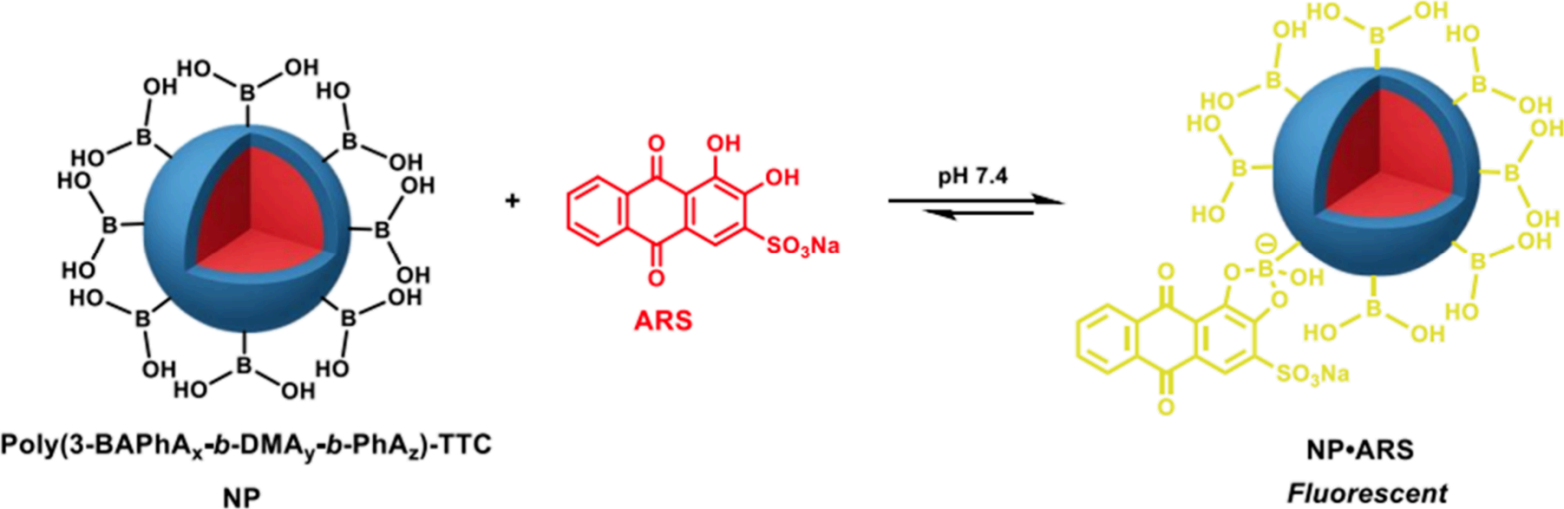
Equilibrium for NP Binding to ARS to give the Fluorescent Complex, NP•ARS

### ARS Binding Studies

For all four NP samples, the intensity of fluorescence increased approximately linearly with the initial concentration of ARS ([ARS]_0_), until an optimal concentration ([ARS]_0_ = ∼50 μM for Runs 3, 5 and 7, and 35 μM for Run 6), from where the signal decreases (Figure 2A). This deviation from linearity is due to nonsteady state conditions being reached, where the BA binding sites become limited. It should be noted however that these [ARS]_0_ concentrations do not reflect the absolute concentration of NP•ARS formed due to difficulties in sampling the same volume of NPs from dispersion Runs 3, 5, 6, and 7.

**Figure 2:**
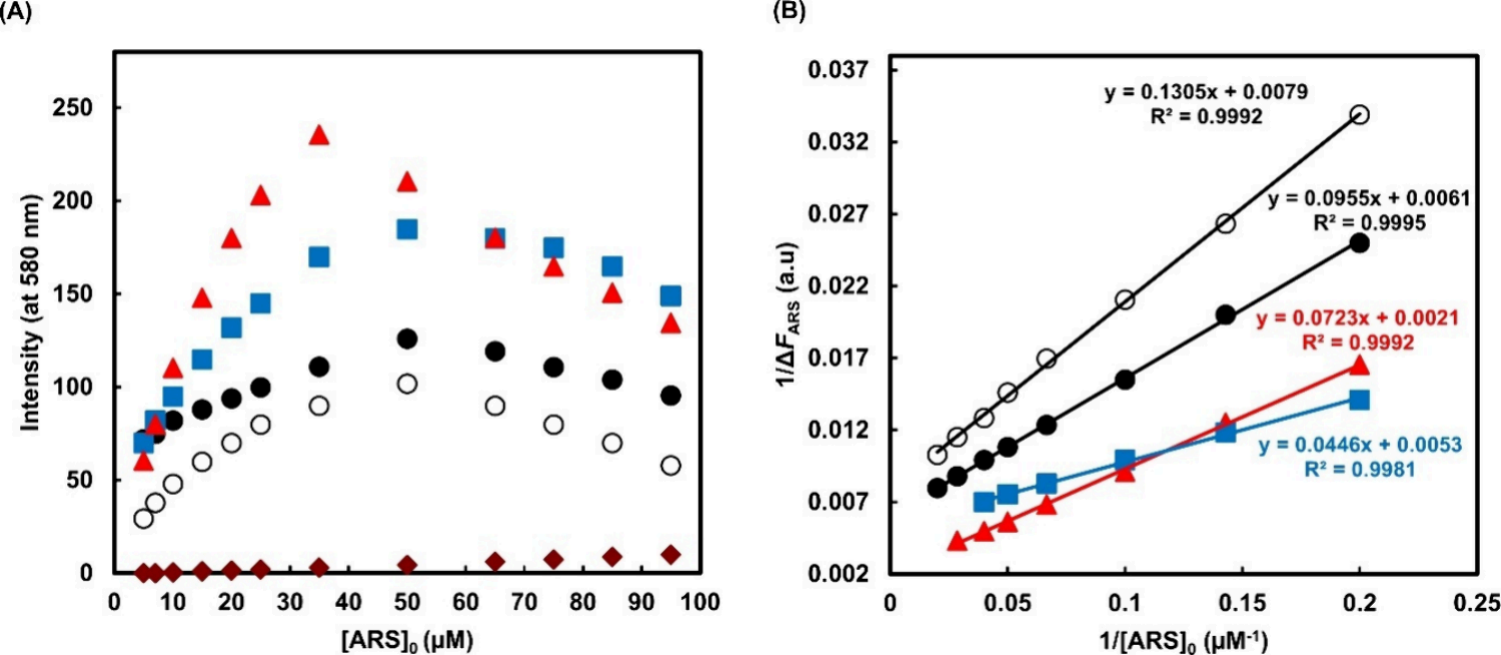
Fluorescence of NP dispersions (∼90 μM) bound to ARS at pH 7.4 after 1 h incubation at room temperature. Excitation at 470 nm. (A) Emission intensity as a function of initial ARS concentration: Run 3 (filled circles), Run 5 (open circles), Run 6 (red triangles), Run 7 (blue squares), and ARS in the absence of NPs (diamonds). Each fluorescence intensity is in triplicate. (B) Benesi–Hildebrand (B–H) plots with lines of best fit.

To determine *K*_ARS_ using the B–H relationship, the concentration of ARS must be much lower than the concentration of available BA sites and the dissociated species should not contribute to the fluorescence signal. Initial ARS concentrations up to the optimal value were used to plot the B–H (Figure 2A), where the contribution of free (unbound) ARS to the fluorescence signal was found to be the recommended <5% (Tables S2–S5 and Figure 2B).^33^

The B–H eq (eq 7) is derived from the equilibrium concentrations (eq 1, Scheme 1),^35^ and is notably different to Brooks et al.,^18^ where the ARS concentration was constant. In our case, the initial ARS concentration [ARS]_0_ is varied in the presence of an excess initial nanoparticle concentration [NP]_0_, and [NP•ARS] originates from [NP]_0_ (eq 2).

$$K_{ARS}=\frac{\left[NP•\mathrm{ARS}\right]}{{\left[\mathrm{NP}\right]}_{0}{\left[\mathrm{ARS}\right]}_{0}}$$ 1

$$K_{ARS}=\frac{\left[NP•\mathrm{ARS}\right]}{\left({\left[NP\right]}_{0}-\left[NP•\mathrm{ARS}\right]\right){\left[\mathrm{ARS}\right]}_{0}}$$ 2

Rearrangement gives eq 3:

$$\frac{{\left[NP\right]}_{0}}{\left[NP•\mathrm{ARS}\right]}=\frac{1}{{\left[\mathrm{ARS}\right]}_{0}K_{ARS}}+1$$ 3

Δ*F*_ARS_ is the change in fluorescence intensity of ARS at 580 nm in the presence and absence of [NP]_0_. Δ*F*_ARS_ represents the formation of NP•ARS (Tables S2–S5 and Figure S5) with due consideration to the constant related to the instrument parameters (*k*), the quantum yield (Φ), the incident light intensity (*I*_0_), the molar absorptivity (ε), and the path length (*b*) (eq 4):

$${\Delta F}_{\mathrm{ARS}}=kfI_{o}\varepsilon b\left[NP•\mathrm{ARS}\right]$$ 4

Rearrangement gives eq 5:

$$\left[NP•\mathrm{ARS}\right]=\frac{{\Delta F}_{\mathrm{ARS}}}{kfI_{o}\varepsilon b}$$ 5

Substituting eq 5 into eq 3 gives eq 6.

$$\frac{kfI_{o}\varepsilon b{\left[\mathrm{NP}\right]}_{0}}{{\Delta F}_{\mathrm{ARS}}}=\frac{1}{{\left[\mathrm{ARS}\right]}_{0}K_{ARS}}+1$$ 6

Both sides of eq 6 are divided by *kfI*_*o*_ε*b*[NP]_0_ to give the B–H eq (eq 7). *K*_ARS_ is determined from the double reciprocal plot of 1/Δ*F*_ARS_ versus 1/[ARS]_0_ by dividing the intercept by the slope, where *k*, Φ, I_0_, ε, and *b* cancel (Figure 2B).

$$\frac{1}{{\Delta F}_{\mathrm{ARS}}}=\frac{1}{kfI_{o}\varepsilon b{\left[NP\right]}_{0}K_{\mathrm{ARS}}{\left[\mathrm{ARS}\right]}_{0}}+\frac{1}{kfI_{o}\varepsilon b{\left[NP\right]}_{0}}$$ 7

*K*_ARS_ for ARS binding to NPs is found to be 63,900 M^–1^ for poly­(3-BAPhA_28_-*b*-DMA_41_-*b*-PhA_500_)-TTC (Run 3), which is approximately 28 times greater than that reported for 3-acetamidophenylboronic acid (3-BAPh)^18^ under similar conditions (Table 2). 3-BAPh has the same chemical structure as a binding unit in the triblock copolymer NP. Further the difference in magnitude between *K*_ARS_ for the NP and 3-BAPh corresponds to the *DP* of the poly­(3-BAPhA) block, so validating our fluorescence assay. There is a marginal decrease in *K*_ARS_ (= 60,500 M^–1^) with NP size, when comparing Runs 3 and 5 composed of the same poly­(3-BAPhA) block, and *DP*s = 500 and 1,500 for poly­(PhA) at the hydrophobic core. Binding can be proportionally varied according to the size of the sensing poly­(3-BAPhA) block, as demonstrated for Runs 6 and 7, with *K*_ARS_ values of 29,050 and 107,800 M^–1^. In these cases, there is a greater discrepancy between B–H and GPC derived *DP*, which is not surprising given inherent errors in GPC, where analysis requires pinacol protection of BA units and *M*_n_ is from calibration against linear poly­(styrene) standards (Table 1).

**Table 2:** Apparent Binding Constant (*K*_ARS_) and Derivation of *DP* for the Poly­(3-BAPhA) Block

Run	B–H, *K*_ARS_ (M^–1^)	*DP* using B–H^a^	*DP* using GPC^b^
3	63,900 ± 80	29	28
5	60,500 ± 65	28	28
6	29,050 ± 50	13	10
7	107,800 ± 75	49	53

a Relative to *K*_ARS_ = 2200 ± 73 M^–1^ for 3-BAPh at pH = 7.4.^18^

b From Table 1. *K*_ARS_ values are reported as mean ± SD, *n* = 3.

### LA Monitoring using FIDA

Displacement of ARS from the fluorescent complex (NP•ARS) with the quencher LA utilized dispersion runs incubated for 1 h at room temperature with known concentrations of LA prepared in the same buffer solutions. Static quenching occurs to give the nonfluorescent boronate ester (NP•LA), where the emission intensity is reduced due to the depreciation in available NP•ARS (Scheme 2 and Figure 3A). To minimize ARS fluorophore interference, NP•ARS dispersions all contained free [ARS]_0_ = < 5%, which are within the range of excess available BA-binding site concentrations (Figure 2A). Run 5 was not investigated, since particle size was found to have minimal effect on binding to BA moieties (see above).

**Scheme 2:**
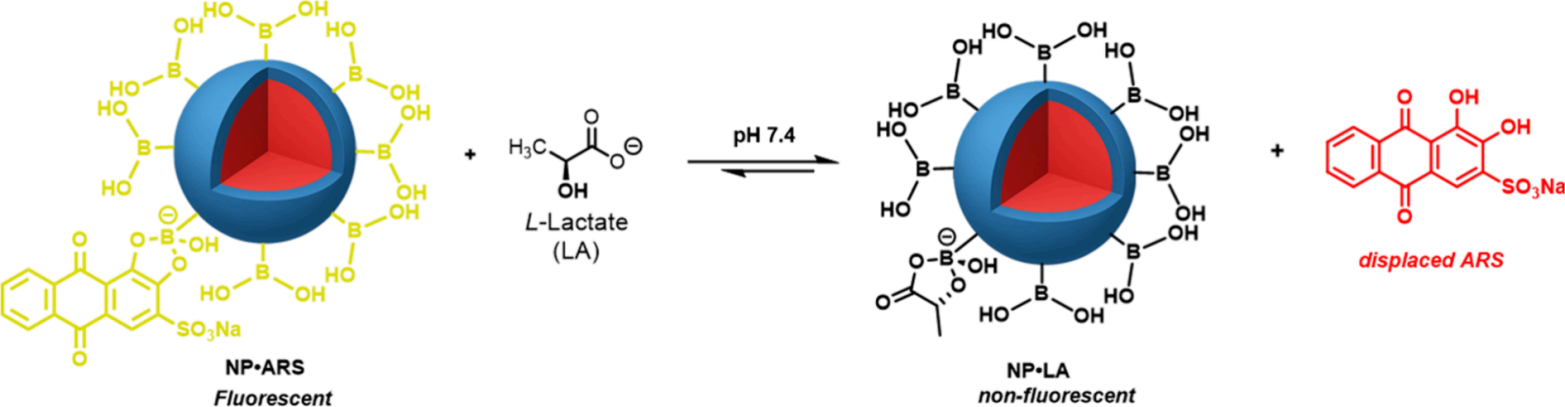
Equilibrium for FIDA: ARS Displacement from NP•ARS with LA to give NP•LA

**Figure 3:**
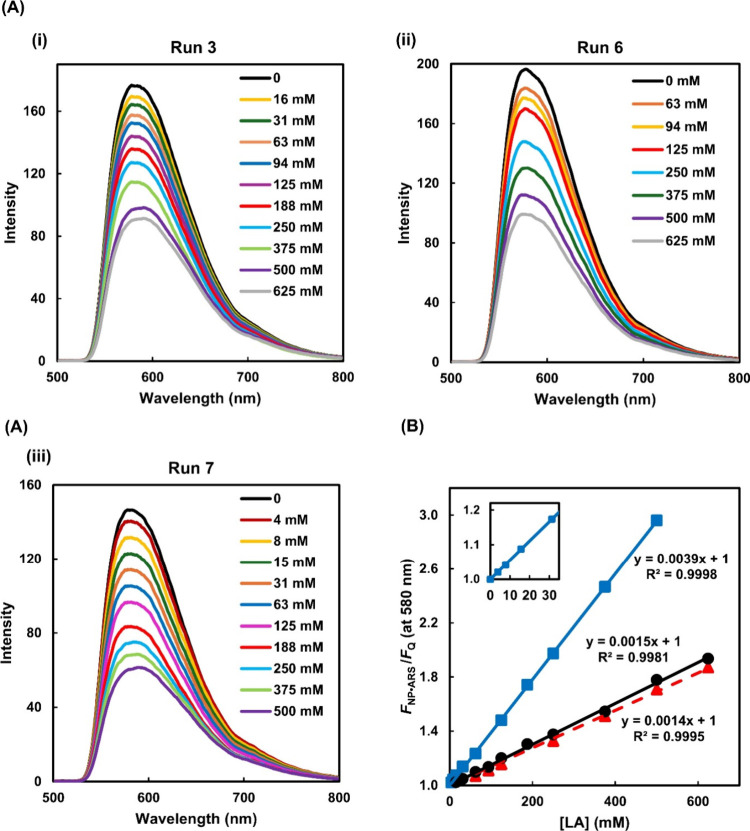
FIDA for LA quenching at pH 7.4 after excitation at 470 nm. NP•ARS generated from Runs 3, 6, and 7 using [ARS]_0_ = 50, 35, and 25 μM, respectively, followed by 1 h incubation with LA at room temperature ([NP]_0_ is different in **(i)**-**(iii)**): (A) Fluorescence spectra with LA concentrations stated within. (B) Stern–Volmer (S–V) plots with lines of best fit for Runs 3 (circles), 6 (triangles), and 7 (squares). Inset is expansion of Run 7 S–V plot. Each fluorescence intensity is in triplicate.

NP•LA and displaced ARS are in equal concentrations and can be calculated via the difference in the fluorescence signal for NP•ARS in the presence and absence of LA, which gives Δ*F*_NP•ARS_ (Tables S6–S8). By independently measuring the emission intensity for free ARS in the presence of the specified [LA], the contribution of the displaced ARS (*F*_(displaced ARS)_) to the fluorescence emission signal (*F*_Q_) was derived. A linear relationship exists between emission intensity and quencher concentration [Q = LA], as given by the Stern–Volmer (S–V) eq (eq 8, Figure 3B).^36^

$$\frac{F_{\mathrm{NP}•\mathrm{ARS}}}{F_{Q}}=1+K_{Sv}\left[Q\right]$$ 8

Where *F*_NP•ARS_ and *F*_Q_ are fluorescence emission in the absence and presence of fluorescence quencher (Q).

The concentration of the second emitter (displaced ARS) is found not to be large enough to affect the linearity of the S–V plot. Upward deviations due to both static and dynamic quenching behaviors and downward deviations due to the presence of two emitters quenching at different rates do not apply to this system,^36^ where the displaced ARS does not bind to the LA quencher. The S–V plots allowed LA monitoring to be carried out up to 625 mM for Runs 3 and 6 and 500 mM for Run 7. At these highest LA concentrations, 16.0–23.5 μM of ARS is displaced and the emission contribution is ∼ 10.4% of the total signal (*F*_Q_) (Tables S6–S8). From the slope of the S–V plots, the equilibrium constant for displacement (*K*_sv_) is derived (Figure 3B). *K*_sv_ (= 1.4–1.5 M^–1^, Table 3) is as expected approximately the same for the two different polymer NP dispersions of Runs 3 and 6 measured under the same conditions (Table 3), since the changes in the fluorescence intensity should be independent of the number of BA sites and dependent upon the binding affinity of BA to LA. *K*_sv_ is however more than 2.5 times greater when using NP dispersion Run 7, presumably because LA monitoring was carried out in 1:1 (pH 7.4) PBS:EtOH, rather than aqueous PBS (pH 7.4) alone. The solvent effect is in line with binding constants for BA-diols reported as 2 orders of magnitude higher in aprotic solvents compared to the protic counterparts.^37^ The higher *K*_sv_ for Run 7 allows lower LA concentrations to be detected due to the use of the alcoholic aqueous solution, with a monitoring range of 4.0–500 mM and limit of detection (*LOD*) of 2.6 mM. *LOD* however varies with [NP]_0_ sampling from the PISA dispersion (*i.e*. the higher the concentration of NPs sampled, the greater *F*_NP•ARS_ or the fluorescence intensity of the blank).

**Table 3:** Summary of Investigated FIDA^a^

Run	Q	*K*_sv_ (M^–1^)^b^	Detection Range (mM)^b^,^c^	Limit of Detection *(LOD*, mM*)* ^d^
3	*L*-Lactate	1.5 ± 0.05	16–625	13.9
6	*L*-Lactate	1.4 ± 0.01	63–625	7.7
7	*L*-Lactate	3.9 ± 0.02	4.0–500	2.6
3	*D*-Glucose	0.4 ± 0.005	50–1000	29.6
3	*D*-Fructose	19.7 ± 0.04	5.0–100	1.1

a PISA dispersions in pH 7.4 PBS solution, apart from Run 7 in 1:1 pH 7.4 PBS:EtOH. NP•ARS incubated for 1 h with quencher at room temperature. Excitation at 470 nm and measured fluorescence at 580 nm.

b Figure 3 and Figure S6.

c Tables S6–S10.

d *LOD* = 3σ/*K*_SV_, where σ represents the fluorescence intensity standard deviation of the blank solution (in the absence of quencher). *K*_SV_ values are reported as mean ± SD, *n* = 3.

Unlike LA, the FIDA using phenylboronic acid with saccharides are well-established.^13–19^ For comparison, displacement assays under the same conditions as for LA, with *D*-glucose and *D*-fructose were carried out using the Run 3 NP dispersion. Using the linear S–V plots, *K*_sv_ is found to be more than 3.5 times smaller and 13 times greater for *D*-glucose and *D*-fructose than LA respectively (Figures S6 and Table 3). The differences in *K*_sv_ values are similar to the difference in association constants for glucose and fructose with 3-BAPh.^18^ BA binding to monosaccharides is dictated by the orientation and relative position of the hydroxyl groups with *D*-fructose solutions known to have the greatest binding affinity due to the high abundance of the *cis*-diol form (β-*D*-fructofuranose).^38^ The low binding affinity for glucose results in low concentrations of displaced ARS at relatively high quencher concentrations ([displaced ARS] = 8.8 mM at [*D*-glucose] = 1 M, Table S9), which contributes ∼ 2.9% emission to the total fluorescence signal at 1 M *D*-glucose. In contrast, the displacement assay is useful at detecting low *D*-fructose concentrations and is utilized between 5.0 and 100 mM (*LOD* = 1.1 mM), with the displaced ARS contribution to fluorescence (*∼*10.5%) at 100 mM comparable to the highest LA concentrations monitored (Table S10).

## Conclusions

BA-functionalized PISA derived core–shell NPs are utilized in the development of FIDA for LA monitoring. Dispersion RAFT polymerization of PhA gave stable and boroxine-free polyacrylamide spherical NPs when using BA-functionalized macroRAFT as part of the hydrophilic solubilized steric-stabilizer block. A B–H approach to ARS binding to NPs enables fluorescence measurement of the *DP* of the BA-substituted block, which applies only to core–shell NPs where the BA moieties are part of the shell. Displacement of ARS from fluorescent NP•ARS by the LA quencher allows the monitoring of LA from 4.0 to 500 mM (*LOD* 2.6 mM) when NPs are dispersed in 1:1 (pH 7.4) PBS:EtOH. The FIDA measures LA beyond the upper limits of current commercial monitors and is potentially useful in investigating elevated LA in various industrial and medical settings, as well as in sweat monitoring of high endurance athletes that can range from 16 to >100 mM.^7^ A limitation of this new technology for LA measurement could be the use of alcoholic solvents, however colloidal dispersions are also achieved using aqueous physiological-like conditions, thus we believe a proof-of-concept demonstration is achieved.

## Supplementary Material



## Notes

Additional experimental details, photographs, GPC, DLS, and TEM images; additional results for binding studies in Figures S1–S6 and Tables S1–S10 (PDF)
