# Correction to “Lactate Monitoring using Fluorescence with Stable Boronic Acid-Functionalized Nanoparticles from Polymerization-Induced Self-Assembly (PISA)”

**DOI:** 10.1021/acs.langmuir.6c03744

**Published:** 2026-06-30

**Authors:** Morvarid H. Balouchi, Zixiao Liu, Fumi Ishizuka, Joseph C. Bear, Hachemi Kadri, Per B. Zetterlund, Fawaz Aldabbagh

The quantum yield symbol should read Φ and not *f* in eqs 4, 5, 6 and 7.

The corrected equations are

4 $${\Delta F}_{ARS}=k\Phi I_{o}\varepsilon b\left[NP\cdot ARS\right]$$

5 $$\left[NP\cdot ARS\right]=\frac{{\Delta F}_{ARS}}{k\Phi I_{o}\varepsilon b}$$

6 $$\frac{k\Phi I_{o}\varepsilon b{\left[NP\right]}_{0}}{{\Delta F}_{ARS}}=\frac{1}{{\left[ARS\right]}_{0}K_{ARS}}+1$$

7 $$\frac{1}{{\Delta F}_{ARS}}=\frac{1}{k\Phi I_{o}{\varepsilon b\left[NP\right]}_{0}K_{ARS}{\left[ARS\right]}_{0}}+\frac{1}{k\Phi I_{o}{\varepsilon b\left[NP\right]}_{0}}$$

Therefore, *f* is replaced by Φ in eqs 4, 5, 6 and 7, and the above correction does not change the derived data including the data provided in Table 2 or any of the accompanying discussions and conclusions in this article.

