# Supplementary material for: Lactate Monitoring using Fluorescence with Stable Boronic Acid-Functionalized Nanoparticles from Polymerization-Induced Self-Assembly (PISA)
Source: Langmuir. 2026 May 11;42(20):14281–9. doi: 10.1021/acs.langmuir.6c01033 (PMC13217619; doi:10.1021/acs.langmuir.6c01033)
Supplement: Supplementary file 1 [file la6c01033_si_001.pdf]

Supporting Information

Lactate Monitoring using Fluorescence with Stable Boronic Acid-Functionalized Nanoparticles from Polymerization-Induced Self-Assembly (PISA)

Morvarid H. Balouchi[a], Zixiao Liu[b], Fumi Ishizuka[b], Joseph C. Bear[a], Hachemi Kadri[a], Per B. Zetterlund[b], Fawaz Aldabbagh[a,]*

[a] Health, Education and Society, Knowledge Exchange and Research Institute (HES KERI) and School of Life Sciences, Pharmacy and Chemistry, Kingston University, Penrhyn Road, Kingston upon Thames, KT1 2EE, United Kingdom

[b] Cluster for Advanced Macromolecular Design (CAMD), School of Chemical Engineering, The University of New South Wales (UNSW), Sydney, NSW 2052, Australia

* Correspondence: f.aldabbagh@kingston.ac.uk

Table of Contents

Figure S1. RAFT dispersion polym. of PhA using Poly(3-BAPhA$_{10}$-*b*-DMA$_{132}$)-TTC (Run 6). *Page S1*

Figure S2. RAFT dispersion polym. of PhA using Poly(3-BAPhA$_{53}$-*b*-DMA$_{54}$)-TTC (Run 7). *Page S2*

Figure S3. TEM images of (A) Run 4 and (B) Run 6 before and after dilution. *Page S3*

Table S1. DLS characterization of spherical NPs upon dilution. *Page S4*

Figure S4. Visual appearance and UV-vis absorption spectra. *Page S5*

Figure S5. Fluorescence spectra of [NP]$_0$ dispersions bound to ARS. *Page S6*

Table S2. Measuring the contribution of free [ARS]$_0$ for Run 3 NP binding. *Page S7*

Table S3. Measuring the contribution of free [ARS]$_0$ for Run 5 NP binding. *Page S8*

Table S4. Measuring the contribution of free [ARS]$_0$ for Run 6 NP binding. *Page S9*

Table S5. Measuring the contribution of free [ARS]$_0$ for Run 7 NP binding. *Page S10*

Table S6. *L*-Lactate FIDA for Run 3. *Page S11*

Table S7. *L*-Lactate FIDA for Run 6. *Page S12*

Table S8. *L*-Lactate FIDA for Run 7. *Page S13*

Figure S6. *D*-Glucose and *D*-fructose. (A) Fluorescence spectra (B) Stern-Volmer plots *Page S14*

Table S9. *D*-Glucose FIDA for Run 3. *Page S15*

Table S10. *D*-Fructose FIDA for Run 3. *Page S16*

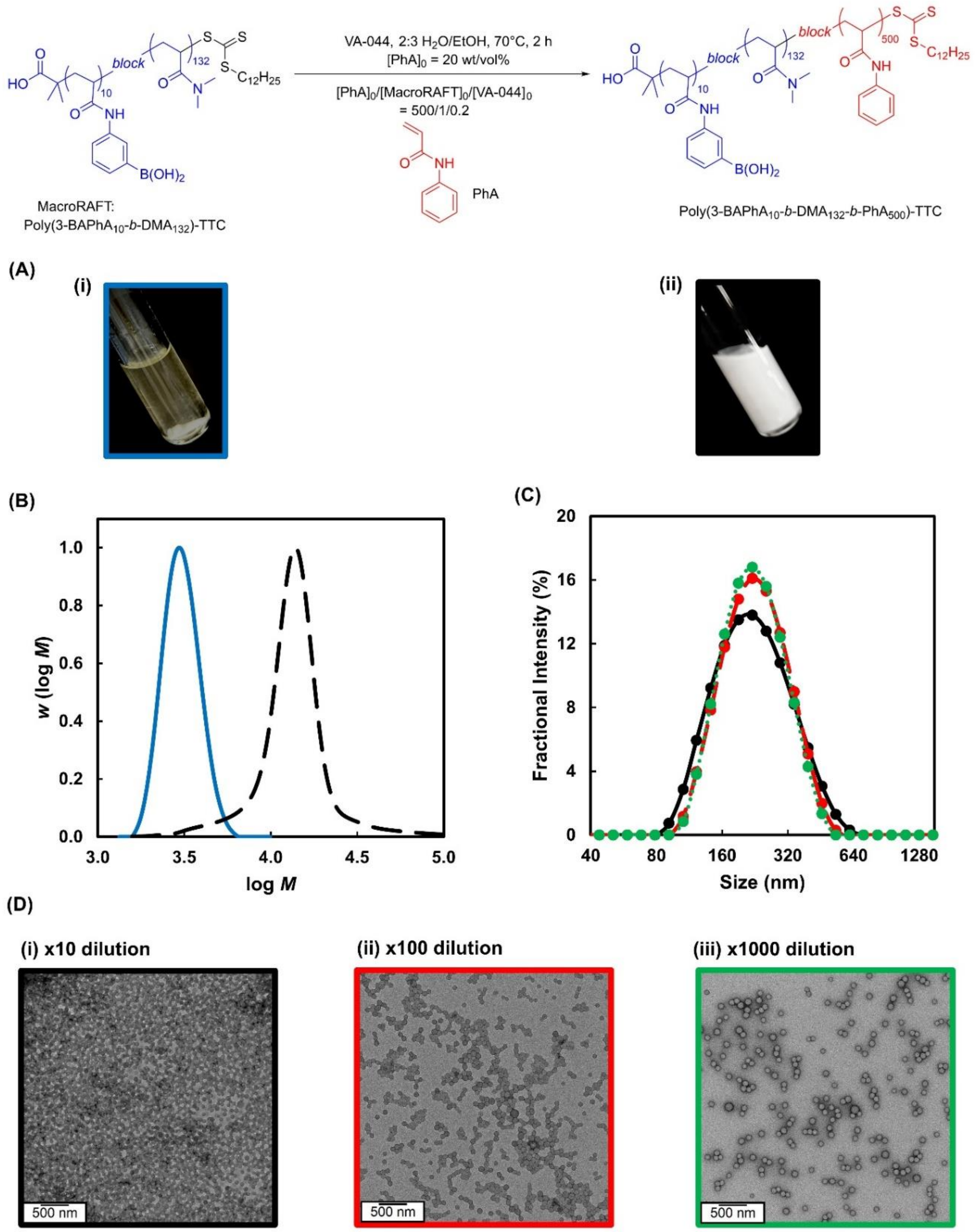

Figure S1. RAFT dispersion polymerization of PhA using Poly(3-BAPhA$_{10}$-*b*-DMA$_{132}$)-TTC (Run 6): **(A)** digital images (including stirrer bar within) **(i)** before and **(ii)** after polymerization; **(B)** GPC after pinacol protection of BA moieties, where the blue continuous line is MacroRAFT; **(C)** DLS after the dilutions in part (D); **(D)** TEM images after **(i)** 10-fold, **(ii)** 100-fold, and **(iii)** 1,000-fold dilution with 2:3 Water/EtOH.

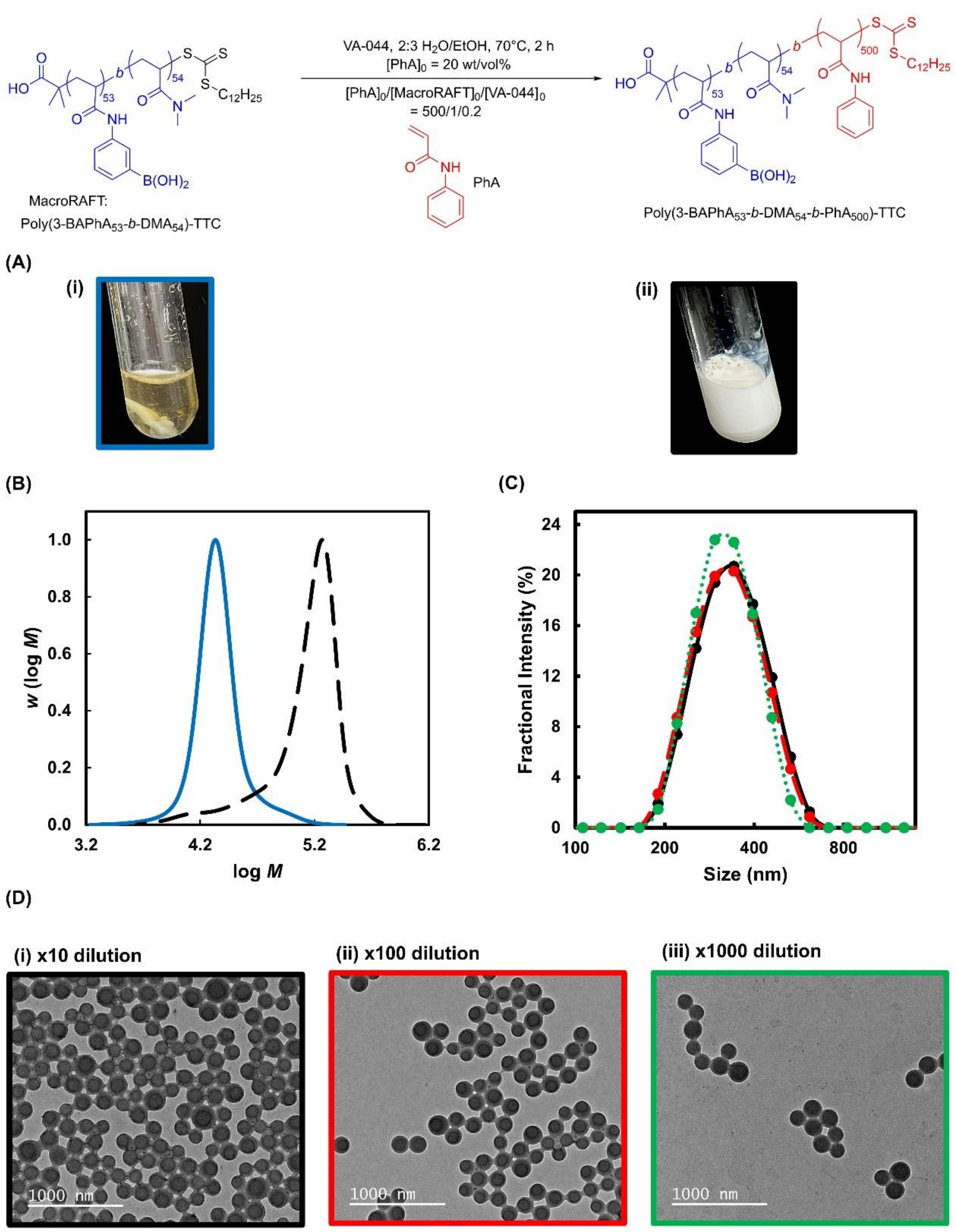

Figure S2. RAFT dispersion polymerization of PhA using Poly(3-BAPhA$_{53}$-*b*-DMA$_{54}$)-TTC (Run 7): **(A)** digital images (including stirrer bar within) **(i)** before and **(ii)** after polymerization; **(B)** GPC after pinacol protection of BA moieties, where the continuous blue line trace is MacroRAFT; **(C)** DLS after the dilutions in part (D); **(D)** TEM images (1.0 μm scale bar within) after **(i)** 10-fold, **(ii)** 100-fold, and **(iii)** 1,000-fold dilution with 2:3 Water/EtOH.

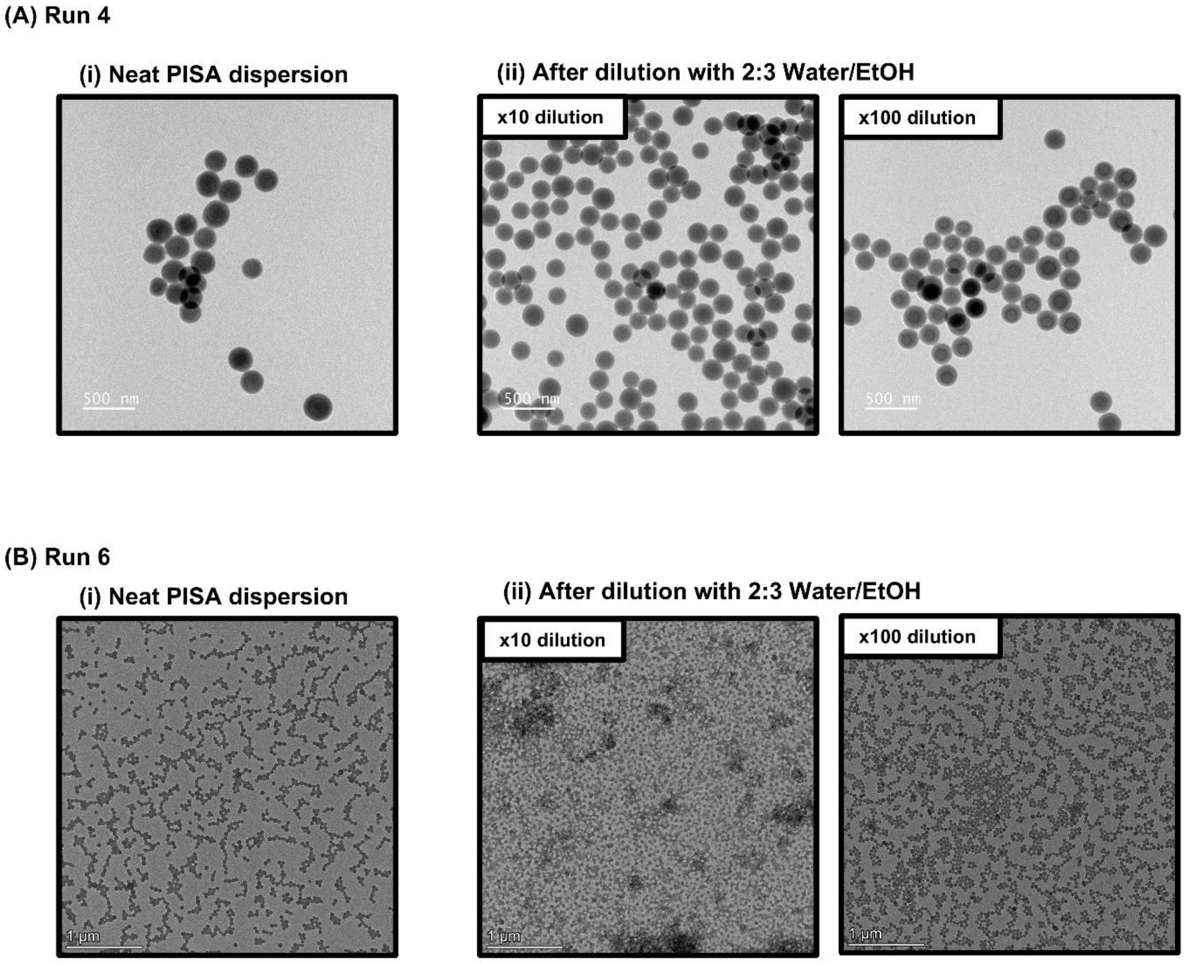

Figure S3. TEM images of **(A)** Run 4 (500 nm scale bar within) and **(B)** Run 6 (1 μm scale bar within): **(i)** neat PISA dispersion; **(ii)** after dilution with 2:3 water/EtOH.

Table S1. DLS characterization of spherical NPs upon dilution with 2:3 water/EtOH at 25 ºC.

Run	Polymer	Dilution	D_h (nm)	PDI	Zeta Potential (mV)
2	Poly(3-BAPhA$_{28}$-*b*-DMA$_{41}$-*b*-PhA$_{250}$)-TTC	x10	337	0.15	-7.2 ± 0.5
2	Poly(3-BAPhA$_{28}$-*b*-DMA$_{41}$-*b*-PhA$_{250}$)-TTC	x100	334	0.07	-7.9 ± 0.8
3	Poly(3-BAPhA$_{28}$-*b*-DMA$_{41}$-*b*-PhA$_{500}$)-TTC	x10	491	0.08	-5.1 ± 0.2
3	Poly(3-BAPhA$_{28}$-*b*-DMA$_{41}$-*b*-PhA$_{500}$)-TTC	x100	510	0.13	-5.4 ± 0.3
4	Poly(3-BAPhA$_{28}$-*b*-DMA$_{41}$-*b*-PhA$_{750}$)-TTC	x10	581	0.04	-4.8 ± 0.1
4	Poly(3-BAPhA$_{28}$-*b*-DMA$_{41}$-*b*-PhA$_{750}$)-TTC	x100	574	0.06	-4.6 ± 0.1
5	Poly(3-BAPhA$_{28}$-*b*-DMA$_{41}$-*b*-PhA$_{1500}$)-TTC	x10	1242	0.09	-5.2 ± 0.8
5	Poly(3-BAPhA$_{28}$-*b*-DMA$_{41}$-*b*-PhA$_{1500}$)-TTC	x100	1171	0.04	-6.4 ± 0.5
6	Poly(3-BAPhA$_{10}$-*b*-DMA$_{132}$-*b*-PhA$_{500}$)-TTC	x10	209	0.17	-4.0 ± 0.7
6	Poly(3-BAPhA$_{10}$-*b*-DMA$_{132}$-*b*-PhA$_{500}$)-TTC	x100	214	0.08	-4.8 ± 0.4
6	Poly(3-BAPhA$_{10}$-*b*-DMA$_{132}$-*b*-PhA$_{500}$)-TTC	x1000	211	0.10	-5.6 ± 0.3
7	Poly(3-BAPhA$_{53}$-*b*-DMA$_{54}$-*b*-PhA$_{500}$)-TTC	x10	325	0.07	-5.1 ± 0.2
7	Poly(3-BAPhA$_{53}$-*b*-DMA$_{54}$-*b*-PhA$_{500}$)-TTC	x100	317	0.05	-5.7 ± 0.3
7	Poly(3-BAPhA$_{53}$-*b*-DMA$_{54}$-*b*-PhA$_{500}$)-TTC	x1000	316	0.08	-5.4 ± 0.2

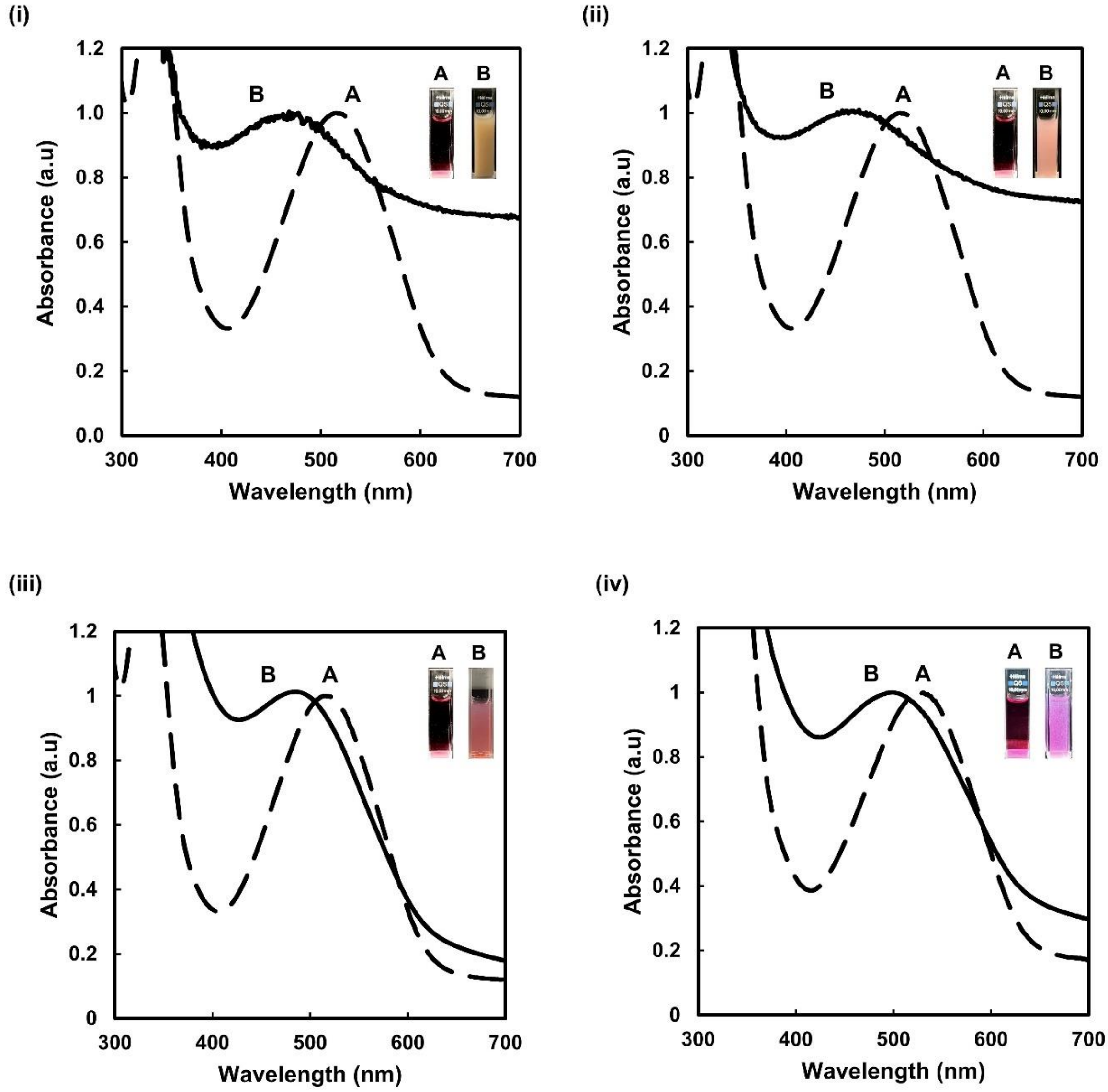

Figure S4. Visual appearance and UV-vis absorption spectra **(A)** Free ARS (50 µM) and **(B)** ARS (50 µM) bound to NPs (~90 µM) after 1 h incubation at room temperature: PISA dispersion **(i)** Run 3; **(ii)** Run 5; **(iii)** Run 6 and **(iv)** Run 7. Runs 3-6 are diluted in pH 7.4 PBS and Run 7 is diluted in pH 7.4 1:1 PBS/EtOH.

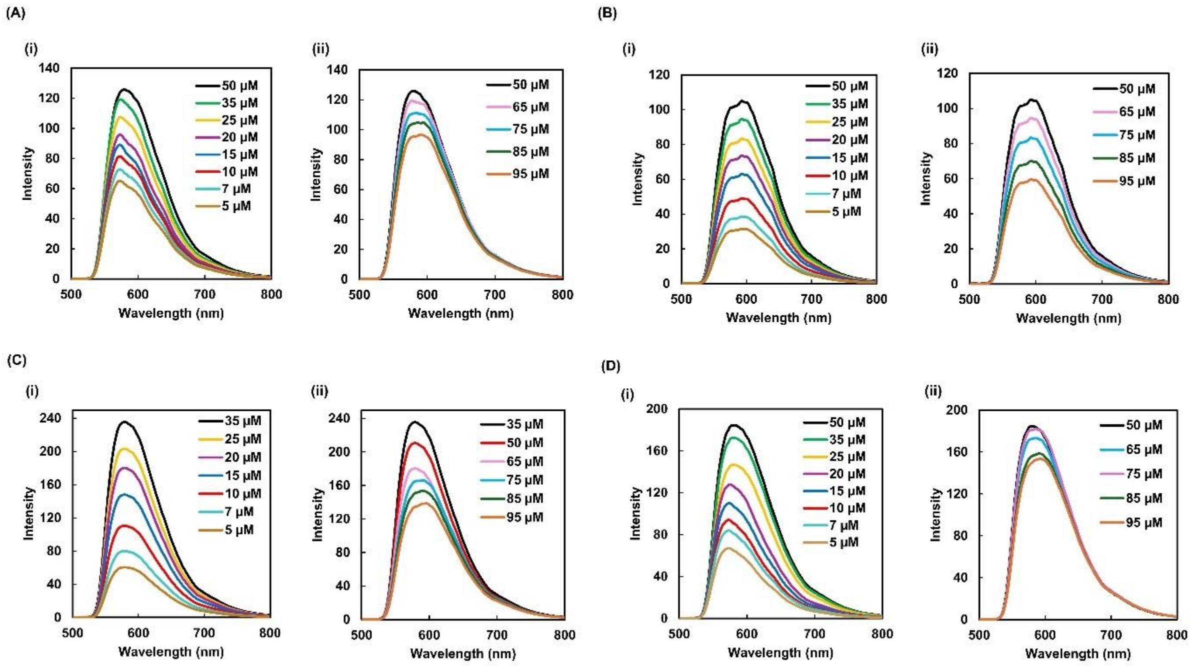

Figure S5. Fluorescence spectra of $[NP]_0$ dispersions (~90 μM) bound to ARS after 1 h incubation at room temperature. Excitation at 470 nm and PISA dispersions diluted with pH 7.4 PBS solution, apart from Run 7 diluted with 1:1 pH 7.4 PBS:EtOH: **(A)** Run 3, **(B)** Run 5, **(C)** Run 6, and **(D)** Run 7 **(i)** increasing and **(ii)** decreasing emission with increasing $[ARS]_0$ (stated within).

Table S2. Measuring the contribution of free $[ARS]_0$ for the binding of ARS to a fixed amount of NP (poly(3-BAPhA$_{28}$-*b*-DMA$_{41}$-*b*-PhA$_{500}$)-TTC) (Run 3) to give NP•ARS in pH 7.4 PBS solution. Excitation at 470 nm.

**$[ARS]_0$ (μM)**	***F***	***F_0***	**% free [ARS]**	**[NP•ARS] (μM)**
5	40	0	0	5.0
7	50	0	0	7.0
10	65	0.5	0.8	9.9
15	82	1	1.2	14.8
20	94	1.5	1.6	19.7
25	103	2	1.9	24.5
35	117	3	2.6	34.1
50	123	4.5	3.7	48.2
65	120	6.2	5.2	61.6
75	110	7.5	6.8	69.9
85	104	8.8	8.5	77.8
95	96	10	10.4	85.1

F is fluorescence intensity at 580 nm of ARS in the presence of NP dispersion. F_0 is fluorescence signal at 580 nm in the absence of NP. % Free [ARS] is calculated from F_0/F x 100%. [NP•ARS] is calculated from $[(F - F_0 = \Delta F_{ARS})/F]$ x $[ARS]_0$.

Table S3. Measuring the contribution of free $[ARS]_0$ for the binding of ARS to a fixed amount of NP (poly(3-BAPhA$_{28}$-*b*-DMA$_{41}$-*b*-PhA$_{1500}$)-TTC) (Run 5) to give NP•ARS in pH 7.4 PBS solution. Excitation at 470 nm.

$[ARS]_0$ (μM)	*F*	F_0	% free [ARS]	[NP•ARS] (μM)
5	29.5	0	0	5.0
7	38	0	0	7.0
10	48	0.5	1.0	9.9
15	60	1	1.7	14.8
20	70	1.5	2.1	19.6
25	80	2	2.5	24.4
35	90	3	3.3	33.9
50	102	4.5	4.4	47.8
65	90	6.2	6.9	60.5
75	80	7.5	9.3	68.0
85	70	8.8	12.6	74.3
95	58	10	17.2	78.7

F is fluorescence intensity at 580 nm of ARS in the presence of NP dispersion. F_0 is fluorescence signal at 580 nm in the absence of NP. % Free [ARS] is calculated from F_0/F x 100%. [NP•ARS] is calculated from $[(F - F_0 = \Delta F_{ARS})/F]$ x $[ARS]_0$.

Table S4. Measuring the contribution of free $[ARS]_0$ for the binding of ARS to a fixed amount of NP (poly(3-BAPhA$_{10}$-*b*-DMA$_{132}$-*b*-PhA$_{500}$)-TTC) (Run 6) to give NP•ARS in pH 7.4 PBS solution. Excitation at 470 nm.

$[ARS]_0$ (µM)	*F*	F_0	% free [ARS]	[NP•ARS] (µM)
5	60	0	0	5
7	80	0	0	7
10	110	0.5	0.5	9.9
15	148	1.0	0.7	14.0
20	180	1.5	0.8	18.4
25	203	2.0	1.0	24.8
35	236	3.0	1.3	34.6
50	211	4.5	2.1	49.0
65	180	6.2	3.4	62.8
75	165	7.5	4.5	71.6
85	151	8.8	5.8	80.1
95	135	10.0	7.4	88.0

F is fluorescence intensity at 580 nm of ARS in the presence of NP dispersion. F_0 is fluorescence signal at 580 nm in the absence of NP. % Free [ARS] is calculated from F_0/F x 100%. [NP•ARS] is calculated from $[(F - F_0 = \Delta F_{ARS})/F]$ x $[ARS]_0$.

Table S5. Measuring the contribution of free $[ARS]_0$ for the binding of ARS to a fixed amount of NP (poly(3-BAPhA$_{53}$-*b*-DMA$_{54}$-*b*-PhA$_{500}$)-TTC) (Run 7) to give NP•ARS in pH 7.4 1:1 PBS/EtOH solution. Excitation at 470 nm.

**$[ARS]_0$ (μM)**	***F***	***F_0***	**% free [ARS]**	**[NP•ARS] (μM)**
5	71	0	0	5
7	85	0.5	0.6	6.6
10	102	1.0	0.8	9.2
15	123	2.0	1.0	14.9
20	137	4.0	2.9	19.4
25	148	5.0	3.4	24.2
35	170	8.0	4.7	33.4
50	184	9.0	4.9	47.6
65	180	10.0	5.0	61.8
75	175	11.0	6.3	70.3
85	159	11.7	7.4	78.7
95	149	12.3	8.3	87.1

F is fluorescence intensity at 580 nm of ARS in the presence of NP dispersion. F_0 is fluorescence signal at 580 nm in the absence of NP. % Free [ARS] is calculated from F_0/F x 100%. [NP•ARS] is calculated from $[(F - F_0 = \Delta F_{ARS})/F]$ x $[ARS]_0$.

Table S6. Fluorescence Indicator Displacement Assay (FIDA) for Run 3. NP dispersion incubated with ARS for 1 h and subsequently incubated for 1 h with *L*-lactate (LA) in pH 7.4 PBS solution at room temperature. Excitation at 470 nm and measured fluorescence intensity at 580 nm. $[NP•ARS]_0$ is different to B-H analysis (above).

**[LA] (mM)**	F_Q	$\Delta F_{NP•ARS}$	**[NP•ARS] remaining (µM)**	**[NP•LA] (µM)**	$F_{(displaced ARS)}$	**% ARS contribution to F_Q**
0	$F_{NP•ARS}$ = 176	0	$[NP•ARS]_0$ = 48.7		0	0
16	172	4	47.6	1.1	1.1	0.6
31	168	8	46.5	2.2	2.0	1.2
63	160	16	44.3	4.4	2.5	1.6
94	155	21	42.9	5.8	3.1	2.0
125	147	29	40.7	8.0	3.6	2.4
188	135	41	37.4	11.3	4.0	3.0
250	128	48	35.4	13.3	6.1	4.8
375	114	62	31.5	17.2	8.3	7.3
500	99	77	27.4	21.3	8.8	8.9
625	91	85	25.2	23.5	9.5	10.4

F_Q is fluorescence in the presence of quencher (LA). $\Delta F_{NP•ARS}$ is the difference in fluorescence in the presence and absence of LA. [NP•LA] = [displaced ARS] is calculated from $\{(\Delta F_{NP•ARS}/F_{NP•ARS}$ at $[LA]_0 = 0)$ x $[NP•ARS]_0\}$. $F_{(displaced ARS)}$ is fluorescence of ARS in the absence of NP at the LA concentrations specified. % Displaced ARS contribution is $\{F_{(displaced ARS)}/F_Q$ at [LA] specified x 100%$\}$.

Table S7. Fluorescence Indicator Displacement Assay (FIDA) for Run 6. NP dispersion incubated with ARS for 1 h and subsequently incubated for 1 h with *L*-lactate (LA) in pH 7.4 PBS solution at room temperature. Excitation at 470 nm and measured fluorescence intensity at 580 nm. $[NP•ARS]_0$ is different to B-H analysis (above).

**[LA] (mM)**	F_Q	$\Delta F_{NP•ARS}$	**[NP•ARS] remaining (µM)**	**[NP•LA] (µM)**	$F_{(displaced ARS)}$	**% ARS contribution to F_Q**
**0**	$F_{NP•ARS}$ = 196	0	$[NP•ARS]_0$ = 34.6	0	0	0
63	184	12	32.4	2.1	2.4	1.3
94	177	19	31.2	3.4	3.0	1.7
125	170	26	30.0	4.6	3.6	2.1
250	148	48	26.1	8.5	5.2	3.5
375	130	66	22.9	11.6	6.2	4.8
500	112	84	19.8	14.8	7.5	6.7
625	90	97	17.5	17.1	9.4	10.4

F_Q is fluorescence in the presence of quencher (LA). $\Delta F_{NP•ARS}$ is the difference in fluorescence in the presence and absence of LA. [NP•LA] = [displaced ARS] is calculated from $\{(\Delta F_{NP•ARS} / F_{NP•ARS}$ at $[LA]_0 = 0)$ x $[NP•ARS]_0\}$. $F_{(displaced ARS)}$ is fluorescence of ARS in the absence of NP at the LA concentrations specified. % Displaced ARS contribution is $\{F_{(displaced ARS)}/F_Q$ at [LA] specified x 100%$\}$.

Table S8. Fluorescence Indicator Displacement Assay (FIDA) for Run 7. NP dispersion incubated with ARS for 1 h and subsequently incubated for 1 h with *L*-lactate (LA) in pH 7.4 PBS/EtOH solution at room temperature. Excitation at 470 nm and measured fluorescence intensity at 580 nm. [NP•ARS]$_0$ is different to B-H analysis (above).

[LA] (mM)	F_Q	$\Delta F_{NP \cdot ARS}$	[NP•ARS] remaining (µM)	[NP•LA] (µM)	$F_{(displaced\ ARS)}$	% ARS contribution to F_Q
0	$F_{NP \cdot ARS}$ = 148	0	[NP•ARS]$_0$ = 24.2	0	0	0
4.0	145	3	23.7	0.5	0.5	0.3
8.0	142	6	23.2	1.0	0.8	0.6
16.5	138	10	22.6	1.6	1.0	0.7
31.0	130	18	21.3	2.9	1.5	1.2
63.0	120	28	19.6	4.6	2.0	1.7
125.0	105	43	17.1	7.0	2.8	2.7
188.0	85	63	14.0	10.3	3.2	3.8
250.0	75	73	12.3	11.9	3.6	4.8
375.0	70	78	11.4	12.8	4.8	6.9
500.0	50	98	8.2	16.0	5.2	10.4

F_Q is fluorescence in the presence of quencher (LA). $\Delta F_{NP \cdot ARS}$ is the difference in fluorescence in the presence and absence of LA. [NP•LA] = [displaced ARS] is calculated from {($\Delta F_{NP \cdot ARS}/F_{NP \cdot ARS}$ at [LA]$_0$ = 0) x [NP•ARS]$_0$}. $F_{(displaced\ ARS)}$ is fluorescence of ARS in the absence of NP at the LA concentrations specified. % Displaced ARS contribution is {$F_{(displaced\ ARS)}/F_Q$ at [LA] specified x 100%}.

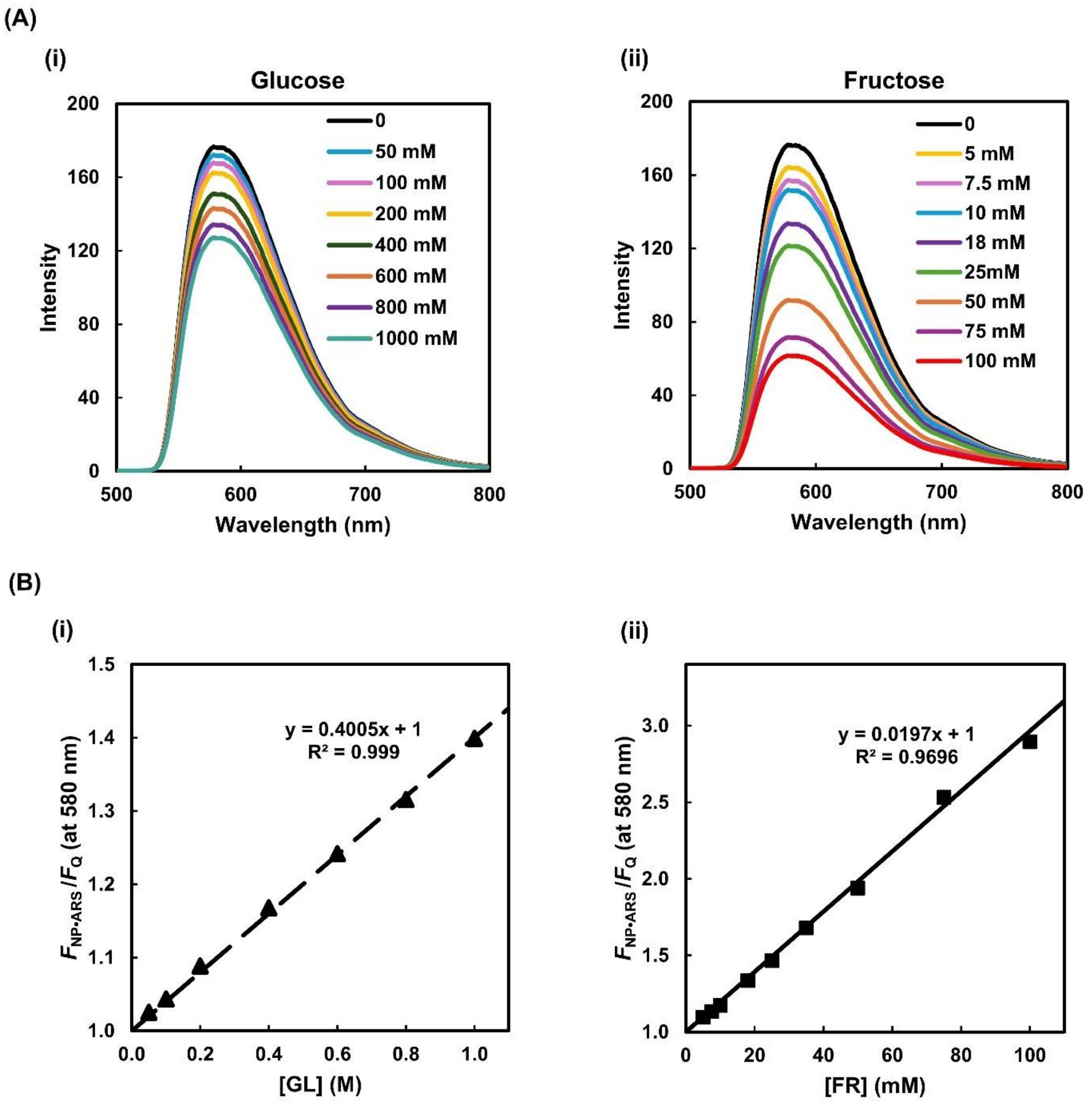

Figure S6. FIDA for quenching using *D*-glucose (GL) and *D*-fructose (FR) at pH 7.4 after excitation at 470 nm. NP•ARS generated from Run 3 using $[\text{ARS}]_0$ = 50 µM, followed by 1 h incubation with quencher at room temperature ($[\text{NP}]_0$ is different in **(i)** and **(ii)**): **(A)** Fluorescence spectra with quencher concentrations stated within. **(B)** Stern-Volmer (S-V) with lines of best fit. Each fluorescence intensity is in triplicate.

Table S9. Fluorescence Indicator Displacement Assay (FIDA) for Run 3. NP dispersion incubated with ARS for 1 h and subsequently incubated for 1 h with *D*-glucose (GL) in pH 7.4 PBS solution at room temperature. Excitation at 470 nm and measured fluorescence intensity at 580 nm. $[NP•ARS]_0$ is different to Run 3 assays (above).

[GL] (mM)	F_Q	$\Delta F_{NP•ARS}$	[NP•ARS] remaining (µM)	[NP•GL] (µM)	$F_{(displaced ARS)}$	% ARS contribution to F_Q
0	$F_{NP•ARS}$ = 176	0	$[NP•ARS]_0$ = 48.7	0	0	0
50	172	4	47.6	1.1	0.8	0.5
100	169	7	46.8	1.9	1.1	0.7
200	162	14	45.3	3.4	1.4	0.9
400	151	25	43.6	5.1	2.1	1.4
600	142	34	42.0	6.7	2.7	1.9
800	135	41	41.0	7.7	3.0	2.2
1000	127	49	39.9	8.8	3.7	2.9

F_Q is fluorescence in the presence of quencher (GL). $\Delta F_{NP•ARS}$ is the difference in fluorescence in the presence and absence of GL. [NP•GL] = [displaced ARS] is calculated from $\{(\Delta F_{NP•ARS}/F_{NP•ARS}$ at $[GL]_0 = 0)$ x $[NP•ARS]_0\}$. $F_{(displaced ARS)}$ is fluorescence of ARS in the absence of NP at the GL concentrations specified. % Displaced ARS contribution is $\{F_{(displaced ARS)}/F_Q$ at [GL] specified x 100%$\}$.

Table S10. Fluorescence Indicator Displacement Assay (FIDA) for Run 3. NP dispersion incubated with ARS for 1 h and subsequently incubated for 1 h with *D*-fructose (FR) in pH 7.4 PBS solution at room temperature. Excitation at 470 nm and measured fluorescence intensity at 580 nm. $[NP•ARS]_0$ is different to Run 3 assays (above).

**[FR] (mM)**	F_Q	$\Delta F_{NP•ARS}$	**[NP•ARS] remaining (µM)**	**[NP•FR] (µM)**	$F_{(displaced ARS)}$	**% ARS contribution to F_Q**
0	$F_{NP•ARS}$ = 176	0	$[NP•ARS]_0$ = 48.7	0	0	0
5.0	161	15	44.5	4.2	1.4	0.87
7.5	156	20	43.2	5.5	1.8	1.2
10.0	150	26	41.5	7.2	2.4	1.6
18.0	132	44	36.5	12.2	3.0	2.3
25.0	120	56	33.2	15.5	3.4	2.8
35.0	105	71	29.1	19.6	3.8	3.6
50.0	91	85	25.2	23.5	4.4	4.8
75.0	70	106	19.4	29.3	5.5	7.9
100.0	61	115	17.2	31.5	6.4	10.5

F_Q is fluorescence in the presence of quencher (FR). $\Delta F_{NP•ARS}$ is the difference in fluorescence in the presence and absence of FR. [NP•FR] = [displaced ARS] is calculated from $\{(\Delta F_{NP•ARS}/F_{NP•ARS}$ at $[FR]_0 = 0) \times [NP•ARS]_0\}$. $F_{(displaced ARS)}$ is fluorescence of ARS in the absence of NP at the FR concentrations specified. % Displaced ARS contribution is $\{F_{(displaced ARS)}/F_Q$ at [FR] specified x 100%$\}$.
